# Barriers and facilitators to implement shared decision making in multidisciplinary sciatica care: a qualitative study

**DOI:** 10.1186/1748-5908-8-95

**Published:** 2013-08-23

**Authors:** Stefanie N Hofstede, Perla J Marang-van de Mheen, Manon M Wentink, Anne M Stiggelbout, Carmen LA Vleggeert-Lankamp, Thea PM Vliet Vlieland, Leti van Bodegom-Vos

**Affiliations:** 1 Department of Medical Decision Making, Leiden University Medical Center, Albinusdreef 2, 2333 Leiden, ZA, The Netherlands; 2 Department of Neurosurgery, Leiden University Medical Center, Albinusdreef 2, 2333 Leiden, ZA, The Netherlands; 3 Department of Orthopedic Surgery, Leiden University Medical Center, Albinusdreef 2, 2333 Leiden, ZA, The Netherlands

**Keywords:** Sciatica, Lumbar radicular syndrome, Implementation strategy, Shared decision making, Barriers and facilitators, Multidisciplinary, Patients, Professionals, Providers

## Abstract

**Background:**

The Dutch multidisciplinary sciatica guideline recommends that the team of professionals involved in sciatica care and the patient together decide on surgical or prolonged conservative treatment (shared decision making [SDM]). Despite this recommendation, SDM is not yet integrated in sciatica care. Existing literature concerning barriers and facilitators to SDM implementation mainly focuses on one discipline only, whereas multidisciplinary care may involve other barriers and facilitators, or make these more complex for both professionals and patients. Therefore, this qualitative study aims to identify barriers and facilitators perceived by patients and professionals for SDM implementation in multidisciplinary sciatica care.

**Methods:**

We conducted 40 semi-structured interviews with professionals involved in sciatica care (general practitioners, physical therapists, neurologists, neurosurgeons, and orthopedic surgeons) and three focus groups among patients (six to eight per group). The interviews and focus groups were audiotaped and transcribed in full. Reported barriers and facilitators were classified according to the framework of Grol and Wensing. The software package Atlas.ti 7.0 was used for analysis.

**Results:**

Professionals reported 53 barriers and 5 facilitators, and patients 35 barriers and 18 facilitators for SDM in sciatica care. Professionals perceived most barriers at the level of the organizational context, and facilitators at the level of the individual professional. Patients reported most barriers and facilitators at the level of the individual professional. Several barriers and facilitators correspond with barriers and facilitators found in the literature (*e.g*., lack of time, motivation) but also new barriers and facilitators were identified. Many of these new barriers mentioned by both professionals and patients were related to the multidisciplinary setting, such as lack of visibility, lack of trust in expertise of other disciplines, and lack of communication between disciplines.

**Conclusions:**

This study identified barriers and facilitators for SDM in the multidisciplinary sciatica setting, by both professionals and patients. It is clear that more barriers than facilitators are perceived for implementation of SDM in sciatica care. Newly identified barriers and facilitators are related to the multidisciplinary care setting. Therefore, an effective implementation strategy of SDM in a multidisciplinary setting such as in sciatica care should focus on these barriers and facilitators.

## Background

Sciatica is a common disorder that is characterized by radiating leg pain in combination with dermatomal motor, sensory, or tendon reflex abnormalities. It is mostly caused by a herniated disc with compression of the nerve root. The prevalence of sciatica in the general population ranges from 1.2% to 43%, depending on its definition [1]. In the Netherlands, most sciatica patients are primarily diagnosed by general practitioners (GPs). A total of 90% of the patients with sciatica recover with conservative therapy [2], with 70% doing so in the first six to eight weeks [3]. Given this favorable outcome during this first period of time, the GP advises to continue daily activities, if necessary with physical therapy and/or pain medication (conservative treatment) when severe neurologic symptoms are lacking. Patients who still suffer from sciatica after six to eight weeks are usually referred to a neurologist for further investigation, including an MRI. If the MRI confirms a herniated disc, the neurologist and patient can consider prolonged conservative treatment or surgery. If they consider surgery, the neurologist can refer the patient to a neurosurgeon or an orthopedic surgeon for the final decision.

A recent randomized controlled trial has shown no significant difference in clinical outcome between conservative treatment and (early) surgery after one or two years [4]. This trial concludes that surgery is more costly but also leads to more rapid relief from the pain, whereas conservative treatment is less invasive [4] but takes patients longer to recover, so that surgery is cost-effective [5]. However this is the only trial that investigated this properly. Other trials are of low quality [4,6]. Because the literature is not convincing about the best treatment option, the choice can be considered preference sensitive [7]. Therefore, the Dutch multidisciplinary guideline recommends that patients and the team of professionals involved in sciatica care jointly decide about treatment (shared decision making [SDM]). In SDM, clinicians and patients make decisions jointly, weighing the evidence regarding different treatment options [8]. In sciatica care, this means that patients are encouraged to consider both treatment options, to communicate their preferences and help select the best treatment for their situation.

Despite the recommendation in the Dutch multidisciplinary sciatica guideline to integrate SDM in consultations [9], there are strong indications that SDM is not yet adopted in clinical practice. Within the Netherlands, surgery rates differ from 31 to 140 per 100,000 inhabitants per region [10]. It is unlikely that this variation is caused by patient preferences or case mix only. Additionally, it has been shown that Dutch patients are used to delegating treatment decisions to their professionals [11]. Part of the variation in surgery rates may thus be associated with preferences of professionals for particular treatment and with a lack of SDM. Given the multidisciplinary nature of sciatica care, SDM has to be integrated in consultations by different professionals at different points in the care process, which may be more difficult than in those cases in which professionals from only one discipline are involved.

To improve SDM implementation, more insight is needed into specific barriers and facilitators of SDM in sciatica care. Previous research concerning SDM implementation mainly focused on one discipline (uni-disciplinary). A systematic review outlines different studies towards barriers and facilitators in uni-disciplinary care [12,13]. Main barriers identified include time constraints and lack of applicability due to patient characteristics or the clinical situation [12]. Main facilitators include motivation of health professionals and the perception that SDM leads to improved patient outcomes and to improved healthcare processes [12]. However, an increasing number of health problems involve multiple disciplines (multidisciplinary care). SDM in multidisciplinary care utilizes the skills and experience of professionals from different disciplines, with each discipline approaching the patient from its own perspective. This mostly involves separate consultations with different professionals [14]. Despite the increase in multidisciplinary care delivery, research into barriers and facilitators for SDM in a multidisciplinary setting, as in sciatica patients, is limited. A previous study that explored barriers and facilitators to SDM focused on barriers and facilitators for integrating SDM in inter-professional (IP) teams, better known as inter-professional SDM (IP-SDM) [15]. Within an inter-professional approach, efforts are made to integrate and translate themes and schemes shared by several professionals [16]. It involves separate disciplines that integrate different approaches mostly into a single consultation [14]. Main barriers related to IP-SDM were an imbalance of power between health professionals of different disciplines, the existence of professional silos, and disagreement about roles and responsibilities between different disciplines [15]. Main facilitators related to IP-SDM were mutual knowledge and understanding of disciplinary roles, trust and respect between different disciplines. Part of these may also apply to multidisciplinary care. However, SDM in multidisciplinary sciatica care involves different disciplines in both primary care and hospital care working independently, who do not see the patient in one and the same consultation, but in several separate consultations [16]. This independent approach within different levels of healthcare may involve other (additional) barriers and facilitators than an inter-professional approach or healthcare that involves professionals working in the same organization. Therefore, the objective of this study is to explore and categorize all barriers and facilitators associated with the implementation of SDM in sciatica care perceived by professionals and patients.

## Methods/Design

To identify barriers and facilitators for SDM in sciatica care, we performed a semi-structured interview study among professionals and a focus group study among patients. Interviews and focus groups reach the parts that quantitative methods cannot reach, because people’s knowledge and attitudes are not entirely encapsulated in reasoned responses to direct questions. This type of data collection can provide rich and in-depth information about the cognitions, motivations and experiences of individuals [17-20], which is well-suited for this type of study. The identification of similarities and differences in perceived barriers and facilitators between professionals and patients contributes to a further understanding of attitudes and beliefs. This is important for the prediction of whether professionals will use SDM, and enables us to develop a tailored-based implementation strategy, with the main goal of improving the use of SDM in daily practice.

### Interviews among professionals

During the period of March 2012 to June 2012, we conducted 40 semi-structured interviews with professionals involved in sciatica care (GP’s, physical therapists [PT], neurologists [NL], neurosurgeons [NS] and orthopedic surgeons [OS] [eight per discipline]) at a location of the participant’s choice (workplace or at home). We applied purposive sampling for the selection of professionals. First, we selected professionals from regions in the Netherlands with high and low surgery rates [10,21], as SDM has been shown to lead to lower surgery rates [22], and we thus would obtain both barriers and facilitators. In addition, we selected professionals working in hospital care in such a way as to ensure diversity of hospital type (general hospitals, university medical centers, and private clinics). The selected professionals received an invitation by e-mail, followed by a telephone call. When professionals did not want to participate, we invited another professional from the same region. To reach the number of 8 professionals for each discipline, we had to approach 8 neurosurgeons (response rate 100%), 10 orthopedic surgeons (response rate 80%), 14 physical therapists (response rate 57%), 16 neurologists (response rate 50%), and 45 general practices (response rate 18%). The most common reasons why professionals did not want to participate were a lack of time or not seeing (many) patients with sciatica in their practice. During the interviews, a topic guide with open-ended questions was used (Additional file 1). The following explanation of SDM was given: ‘In SDM, clinicians and patients make decisions jointly, weighting the evidence regarding different treatment options [8]. In sciatica care this means that patients are encouraged to consider both conservative and surgical treatment options, to communicate their preferences and help select the best treatment for their situation.’ In addition, professionals were asked to give an example of SDM in daily practice to determine whether the explanation was clear enough. Participating professionals received a hundred euro gift card as an incentive. The average duration of an interview was one hour and all interviews were audiotaped and transcribed in full. Interviews were conducted by one of two trained interviewers (SH and MW). Both interviewers have a master’s degree in health sciences. Their education included training in the conduct of interviews and focus groups. The interviewers had no involvement in patient care, and the participants had no personal background information on the interviewers. We continued with interviews until data saturation was reached. Data saturation was reached when no ideas emerged during three consecutive interviews [23].

### Focus groups

In June 2012, we performed three focus group interviews (six to eight patients per group [17]) at the Leiden University Medical Center with patients who had been diagnosed with sciatica within the previous two years. The focus group procedures of Morgan *et al*. [24] were used in preparing and conducting the focus group sessions. We created three homogeneous groups to move patients more quickly to a discussion [24]. One focus group included patients who had had surgery, one included patients who had had conservative treatment, and one focus group included patients who still had to decide on treatment. Patients were recruited via advertisements in local newspapers. Participants ≥18 years, and with a written informed consent were included in the study. Patients with an inability to understand written and oral Dutch instructions were excluded. Patients received a twenty euro gift card as an incentive, and travel costs reimbursement.

Before the focus groups, participants received an information letter. They were asked to think about the decision making process for the treatment of their sciatica before attending the focus group. During the focus groups, a topic guide was used (Additional file 2). We explained the concept of SDM and gave an example of SDM in sciatica care. Participants were asked to write their positive and negative aspects about the decision making process on post-its, and posted these on separate boards. We used these post-its to stimulate discussions between participants. A trained moderator (SH) and an observer (MW) conducted the focus groups. The focus groups lasted two hours, including a 15-minute break. All focus-groups were audiotaped and transcribed in full.

### Analysis

Directed content analysis was used to analyze the interviews and focus groups. This method is well suited for research that would benefit from further description and to extend conceptually a theory or framework [25]. We used the framework of Grol and Wensing [26]. This framework describes how barriers and facilitators can be identified, categorized, and used for the development of a tailored-based intervention strategy to facilitate desired change, in this study implementing SDM [26]. Based on several theoretical reflections on behavioral change, this framework categorizes barriers and facilitators into six levels: the innovation (in our case SDM), the individual professional, the patient, the social context, the organizational context, and the external environment (political and economic factors). We used predetermined barriers/ facilitators of the framework of Grol and Wensing [26] to ensure that we would find all barriers and facilitators for the implementation of SDM in sciatica care. New codes were created for text that could not be categorized within these predetermined barriers/ facilitators. Two researchers (SH and MW) independently coded the interviews and focus groups. Discrepancies were discussed until consensus was reached. In the next step, reported barriers and facilitators were classified according to levels of the framework of Grol and Wensing. After classification of barriers and facilitators within the levels of the framework, three researchers (SH, PM, and LB) independently grouped the barriers and facilitators into themes for comparison between patients and professionals. Discrepancies were discussed until consensus was reached. Participants did not receive feedback on the findings. The software package Atlas.ti 7.0 [27] was used for analysis.

### Ethical approval

This study protocol was presented to the Medical Ethical Committee of the Leiden University Medical Center. An exemption was obtained, as ethical approval for this type of study is not required under Dutch law.

## Results

### Characteristics of the population

Table 1 shows the characteristics of the professionals who participated in the semi-structured interviews. The participating professionals covered a wide range with respect to age, experience and number of patients treated annually. Twenty-two patients participated in the focus groups. Eight patients per focus group were invited; two participants did not show up. Characteristics of the patients are described in Table 2. Participating patients covered a wide range with respect to age and time since diagnosis.

**Table 1:** Characteristics of interviewed professionals

**Discipline**	**n**	**Average age, years (range)**	**Male (%)**	**Average work experience, years (range)**	**Average no. of sciatica patients treated per year (range)**
Physical therapist	8	47 (30–58)	4 (50)	23 (8–33)	56 (6–240)
General practitioner	8	49 (32–63)	5 (63)	17 (1–34)	20 (3–52)
Neurologist	8	49 (37–62)	6 (75)	11 (3.5-22)	311 (52–780)
Neurosurgeon	8	50 (38–62)	6 (75)	16 (5–27)	692 (300–1,404)
Orthopedic surgeon	8	52 (40–67)	8 (100)	16 (4–27)	444 (3–1,300)

**Table 2:** Characteristics of patients in focus groups

**Focus group**	**n**	**Average age, years (range)**	**Male (%)**	**Average time since diagnosis, months (range)**
1. Surgery	8	51 (19–81)	2 (25)	6 (1–18)
2. Conservative therapy	8	56 (19–75)	3 (38)	9 (1–24)
3. Still had to decide	6	51 (33–75)	2 (33)	9 (3–24)

### Barriers and facilitators

We identified 53 barriers and 5 facilitators perceived by professionals (Additional file 3: Table S1) for the implementation of SDM in sciatica patients. These barriers and facilitators could be grouped into 15 themes (Table 3). Professionals perceived most barriers at the level of the organizational context, and facilitators at the level of the individual professional (Additional file 3: Table S1). During the focus groups, 35 barriers and 18 facilitators for SDM on 15 themes (Table 3) were reported by patients regarding their decision making for sciatica treatment (Additional file 4: Table S2). Patients mentioned most barriers and facilitators at the level of the individual professional (Additional file 4: Table S2). Table 3 shows the themes influencing SDM in sciatica care for both professionals and patients. It is clear that more barriers than facilitators were mentioned, particularly by professionals. We will discuss each theme, and which specific barriers and facilitators that were mentioned within these themes.

**Table 3:** Themes influencing SDM in sciatica care according professionals and patients

**Level**	**Theme (Professionals)**	**B**	**F**	**Theme (Patients)**	**B**	**F**
Innovation (SDM)	Unclear concept of SDM	X				
Individual professional	Poor professional-patient relationship	X		Professional- patient relationship	X	X
	Negative (B)/ positive (F) professional’s attitude/ behavior toward SDM	X	X	Negative (B)/ positive (F) professional’s attitude/ behavior towards SDM	X	X
	Lack of knowledge of the professional about SDM/ treatment options	X		Lack of knowledge of the professional about SDM/ treatment options	X	
				Lack of (B)/ sufficient (F) information provision/ explanation	X	X
Patient	Negative patient’s attitude towards SDM	X		Negative (B)/ positive (F) patient’s attitude towards	X	X
	Lack of patient’s capabilities to decide	X		SDM Lack of (B)/ sufficient (F)	X	
	Pressure by patient toward professional	X		patient’s capabilities to decide		
				Lack of knowledge of patient about treatment options	X	
Social context	Lack of inter-professional collaboration	X		Lack of (B)/ sufficient (F) inter-professional collaboration	X	X
	Social influences of third parties	X		Social influences of third parties	X	
Organizational context	Lack of tools to facilitate SDM	X		Lack of (B)/ sufficient (F) tools to facilitate SDM	X	X
	Situational factors (*e.g*., lack of time)	X		Situational factors (*e.g*., lack of time)	X	
	Long waiting list influences decision process	X		Long (B)/ short (F) waiting list influences decision process	X	X
	Poor logistics/ implementation	X		Conflicting information about treatment options	X	
External environment	Environmental influences on the decision process	X		Environmental influences on decision process	X	X
	Reimbursement in favor of surgery	X		Reimbursement in favor of surgery	X	

*B* barrier, *F* facilitator.

### Innovation (SDM)

Professionals mentioned the unclear concept of SDM as a theme. The lack of clarity of the concept of SDM was regarded as a barrier for SDM. With respect to the definition of SDM, many professionals thought they were using SDM. However, when discussing SDM they wondered whether they really met all the conditions (*e.g*., information provision of both treatments’ options, ask patient’s preferences) for a decision to be a shared decision. (OS3: ‘Which conditions do you have to meet before you can say this is decision that has been taken jointly? That is not clear to me’).

### Individual professional

Both professionals and patients mentioned the three themes at this level of the framework: (poor) professional-patient relationship, professional’s attitude/ behavior towards SDM, and lack of knowledge about SDM/ treatment options of professional. In addition, patients mentioned lack of information provision/ explanation by the professional as a theme.

Regarding the first theme, professionals said that a poor professional-patient relationship is a barrier for the SDM process. The relationship may be influenced by the multidisciplinary care patients receive, as they have superficial contacts with multiple professionals, instead of visiting one professional who really knows the patient. Especially professionals in primary care experience difficulties in applying SDM when they are not familiar with the background and personal situation of the patient or when they have a poor relationship with the patient. This may be due to how professionals in primary care in general have a better knowledge about the background and personal situation of most of their patients compared to professionals in hospital care. General practitioners said they have more and more patients in their practices, which makes it more difficult to really know their patients than before when practices were smaller. As a consequence, they experience more difficulties with applying SDM to patients they do not know, while professionals in hospital care are used to dealing with this lack of unfamiliarity. (NL3: ‘You should really know the patient to respond better to the factors playing a role in deciding whether or not the patient needs a surgery. Who knows the patient nowadays?’). Patients also mentioned the importance of a patient-professional relationship, as a barrier and facilitator (bad versus good relationship). For example, they mentioned that some professionals had a lack of attention for their anxiety, personal situation, and preferences, while the elicitation of patient preferences is crucial to SDM. (P3: ‘I had to impose my own will, and with a lot of difficulties the neurologist finally referred me to a surgeon, but I really had to push it through. The neurologist tried to stop me, whereas I had complaints for more than a year without any improvement’).

Another theme is the attitude/ behavior towards SDM. Professionals felt it is important to express their own view about which treatment option to follow, and to determine the next step in the care trajectory, rather than the patient. (OS8: ‘I am not a populist, I am not going to say “Oh this is what you want, you name it, we’ve got it”’). In addition, many professional had an explicit preference for conservative treatment or for surgery. This preference could influence SDM, if professionals push patients towards the treatment of their preference. (OS3: ‘As long as there are no neurological symptoms, no cauda equina syndrome, then of course you do nothing. In those cases you try to convince patients of not having a surgery’). Patients confirmed that some professionals have a strong preference for one of the treatment options and mentioned that professionals tried to push them into the direction of their preference. (P6: ‘My doctor insisted me to wait, to let my body recover by itself’). On the other hand, some professionals had a positive attitude towards SDM. For example, they said that SDM improves quality of care and patient outcomes, which may function as a facilitator for SDM.

The third theme is lack of knowledge of the professional about SDM/ treatment options. Patients felt that some professionals had a lack of knowledge about treatment options, especially in primary care. Professionals frequently told patients that there is only one treatment option. Again, this may be related to the complex structures in the multidisciplinary sciatica setting. Since many professionals are involved, professionals are likely to provide information regarding the treatment they can provide themselves, but have a lack of knowledge about other possible treatment options. (P6: ‘I went to the PT and GP and they said: “Nowadays doctors do not perform sciatica surgeries anymore, you will just have to wait, because your body will recover your herniated disc itself’). The sciatica guideline recommends that the patient and professional together decide on surgical or prolonged conservative treatment after considering the harms and benefits of each treatment option. This is impossible if not all the professionals are familiar with these options or with the sciatica guideline, and thus with the need for SDM in sciatica patients. (NS2: ‘I am not really a guideline person’).

A number of patients received the wrong diagnosis. Due to this wrong diagnosis, patients were suffering from sciatica for a long period of time. It sometimes took weeks or even months before they got the right diagnosis. As a result, the first six to eight weeks of conservative therapy had already passed, and they were referred to hospital care for surgery without given information about the care trajectory or alternative treatment options. The issue of not receiving SDM was thus a consequence of getting the wrong diagnosis. (P22: ‘My GP though there was something with my Achilles tendon or muscles, but it appeared to be an herniated disc’). Furthermore, some professionals perceived a lack of education and skills for SDM, especially communicative skills. (NL4: ‘You need some communication skills, and that is difficult. (…) Communication with the patient is the most important thing. Until now, there is not enough attention for communication skills’).

Patients also mentioned the theme of information provision and explanation, and thought that there is room for improvement concerning this theme. They perceived a lack of information provision with regard to treatment options and potential harms and benefits. (P6: ‘My doctor advised me to wait, and only told me about the disadvantages why I shouldn’t have a surgery. In the end I needed a surgery, but the only thing I could think of were all the disadvantages of having a surgery’). Some patients received sparse information about one of the treatment options. Others did not mention one of the treatment options at all. They also mentioned a lack of explanation by professionals of the care trajectory. (P10: ‘I went to the hospital, they gave me little explanation and no deliberation. They told me: you have a herniated disc, here you have morphine and you can go home now’). Besides these barriers, patients also mentioned facilitators regarding this theme. Most facilitators were in the opposite direction of the reported barriers (*e.g*., sufficient information provision, explanation about harms and benefits of each treatment option, and explanation of the care trajectory).

### Patient

At the level of the patient, both professionals and patients mentioned the attitude/ capabilities of patients. Furthermore, professionals mentioned pressure by patients, and patients mentioned their own lack of knowledge about treatment options.

Regarding the first theme of negative attitude toward SDM/ patient’s capabilities to decide, professionals stated that some patients preferred a professional-dominated over a shared approach. They think that patients do not want to decide together but want to leave the decision up to the professional. (NS7: ‘Not everybody wants a shared decision. Some people want a decision made by the doctor’). In the focus groups, one patient preferred a physician-dominated decision. The other patients preferred a shared decision. (P2: ‘I prefer to make the final decision, it is my body’).

The second theme for professionals is pressure by patients toward professionals. Professionals mentioned that some patients are demanding. Demanding patients are not willing to wait, and put pressure on the GP’s to refer early. Therefore, specialists are seeing patients during their first weeks with sciatica, and patients are demanding an MRI. In this first period, conservative treatment is recommended in the sciatica guideline. Often specialists order an MRI, but in the end, many patients recover during this first period, and the MRI at the hospital was unnecessary. (OS3: ‘Nowadays, patients are not willing to wait for six weeks. Everybody wants an MRI as soon as possible’).

The second theme for patients is their own lack of knowledge about treatment options. Patients said that they did not have enough knowledge to make the final decision. This reflects the information provision and explanation mentioned as barriers at the level of the individual professional, which was mentioned before. (P1: ‘I did not tell my GP that I wanted surgery, because I did not know that was a possibility’).

### Social context

Themes mentioned by both patients and professionals at the level of the social context are (lack of) sufficient inter-professional collaboration and social influences of third parties. Most barriers mentioned are related to the multidisciplinary setting in sciatica care.

Regarding the (lack of) sufficient inter-professional collaboration, professionals found it difficult to get into contact and communicate with each other, especially medical and paramedical professionals. (PT2: ‘Actually, we professionals are all doing our job on our own “island.” We do not have direct contact with each other’). Some patients perceived a good communication between professionals, and said the information exchange between different disciplines made the decision making easier, so that it becomes a facilitator for SDM.

Other patients perceived a lack of communication between professionals. They visited multiple professionals during their care trajectory but had to tell their story many times. They thought it would help if professionals shared information with each other. (P9: ‘If my PT sends a letter to the GP, she does not get an answer. There was also a lack of communication between the medical professionals I visited. It is annoying if you visit a medical professional and there has been no communication at all with the medical professional you have visited previously’).

Besides the lack of communication, there is also a lack of trust in the expertise of other disciplines. Some professionals think that other (para) medical professionals do not have enough knowledge about sciatica or do not inform or treat patients in the right way. Therefore, some professionals do not refer patients, but give patients the treatment they can provide themselves. (NS3: ‘Despite the fact that the neurologist says he informs the patient about conservative treatment, it always is a surprise for patients that natural recovery is a possibility in sciatica’).

Within the theme social influences of third parties, a barrier perceived by professionals was the promotion of one of the treatment options by third parties (*e.g*., professional association). Patients perceived social pressure of family or friends, who sometimes have an outspoken opinion about which treatment the patient should follow.

### Organizational context

Themes mentioned by both patients and professionals at the level of the organizational context were tools to facilitate SDM, situational factors and (long/ short) waiting lists that influence the decision process. In addition, professionals mentioned the poor logistics/ implementation, and patients mentioned conflicting information.

Tools to facilitate SDM were mentioned by both professionals and patients. Despite the availability of two decision aids, professionals mentioned a lack of tools to inform patients as a barrier. Patients mentioned conflicting information in leaflets as a barrier. Tools mentioned as facilitators were access to the professional if the patient wants to change treatment, and the possibility of having a telephone consultation.

The second theme concerns situational factors. Lack of time was mentioned by both professionals and patients. Many professionals perceived a high workload. The time of a consultation ranged from 10 minutes to 45 minutes in public and private hospitals. Professionals with little time said they did not have enough time to discuss everything with the patient, besides the diagnosis of sciatica. Patients also perceived this lack of time. (OS7: ‘I think the factor time is the biggest bottleneck’).

Financial interest is another example of a barrier mentioned by professionals within this theme. In some hospitals, specialists felt they could not apply SDM because they had to reach certain production rates. Some specialists also stated that sciatica surgery is interesting for hospitals because the costs of surgeries are lower than the reimbursement they receive. Therefore, hospitals sometimes reserve the operating rooms for sciatica surgeries. (OS4: ‘For the hospital it is of financial interest that sciatica patients get surgery instead of conservative treatment, so hospitals prefer sciatica surgeries’).

Another theme was the (long/ short) waiting lists that influence the decision making process. Short waiting lists were mentioned by patients as a facilitator. On the other hand, long waiting lists for a hospital visit or surgery was mentioned by both professionals and patients as a barrier. These waiting lists influence the decision making process; for example, some surgeons make the decision (surgery yes or no) based on the length of the waiting list. As a result, the patient is not presented with all options and thus will not have a shared decision. Other professionals already put the patient on the waiting list, just in case the patient should need a referral in the future, and thereby patients miss a step (referral or not) in the decision making process. This referral is not a shared decision, but the decision of the involved professional. (GP4: ‘The neurologists in this region have an enormous waiting list. Sometimes that influences your way to get things done, for example you refer the patient early in the process, so that at least the appointment has already been made’). In addition, some patients said that once they had made the decision to have surgery, they had to wait for a long period of time, whereas the trajectory from primary to hospital care had already taken weeks, or sometimes months. Once the decision for surgery was made, they did not want to suffer pain any longer. (P6: ‘I had to wait for five weeks until I could visit the neurologist, and then another eight to nine week for a surgery. (…) Ultimately you have your surgery, but you are exhausted and the healing process stagnates’).

Professionals mentioned the theme of poor logistics/ implementation of SDM as a barrier. Especially in primary care, there is a lack of clear criteria for referral and/ or surgery, probably associated with multiple disciplines being involved. For instance, some professionals did not know when patients were eligible for surgery, and thus in which situations they can refer patients, offer patients different options for treatment, and can use SDM. (PT1: ‘It would be great if I had clear criteria when to refer the patient to the GP because patients do not need a referral for physical therapy and some have not seen a GP’).

Furthermore, there is a lack of visibility into what other disciplines can do in sciatica care. Professionals said that if they had more insight in what other disciplines can do, they can better explain all the options to the patient, and would be more open to referrals. (NS7: ‘Sometimes anesthesiologists are saying, “you just perform surgeries, but one injection and the pain is gone,” so to speak, but I do not know everything they can do, and that is inadequate’).

Patients reported conflicting information given by different professionals as a barrier. Some patients said they did not know which option they had to choose, because of conflicting information from professionals. In one case, a specialist advised surgery, and another professional advised conservative treatment. In addition, sometimes advices given to patients during the conservative treatment are conflicting as well. (P9: ‘My PT said that it was important to be active, while the GP said I should not move a lot’).

### External environment

With regard to the external environment, professionals and patients both mentioned the themes of reimbursement in favor of surgery and environmental influences on the decision process.

Persons in the Netherlands have a basic insurance package and have the option of purchasing supplementary insurance for additional healthcare. The first nine visits to the physical therapists are included in the basic insurance package. If a patient does not have an optional complementary insurance, they have to cover the cost for the other visits themselves. For some patients, this is a reason to quit their physical therapy and to look for other possibilities. In these cases, professionals referred patients earlier to hospital care, to get surgery. (P2: ‘I will quit physical therapy as soon as I have to pay for it.’ PT8: ‘I can imagine that patients rather have surgery when they do not have a complementary insurance and have to pay for physical therapy’).

Unreliable and conflicting information on treatment options on the internet also hindered both professionals and patients in SDM. Patients read wrong information on the internet, which influenced their treatment or relationship with their caregiver. (P20: ‘I read on the internet about a method in China, where they attach a pole to your back, so you can’t move, but my PT didn’t want to do that’). Professionals also found it time-consuming to talk with patients about all the incorrect information their patients read, while they are already struggling with the factor time. They also said patients would have more anxiety because of all the negative stories they read, which makes it more difficult for the patient to make a well-balanced decision. (GP3: ‘The point is that especially doom diagnoses and complicated courses predominate on the internet and people cannot always correctly apply these to their personal situation’). Therefore, some patients suggested making one website with reliable information about sciatica.

## Discussion

This study addresses several gaps in the literature on SDM. It identifies a large number of barriers and facilitators related to SDM in sciatica treatment, and provides new insights, particularly for multidisciplinary care. To our knowledge, no previous study has focused on barriers and facilitators for SDM in multidisciplinary care trajectories that involve both primary care and hospital care. This multidisciplinary setting, with each discipline approaching the patient from its own perspective in different consultations, makes SDM more complex. We identified barriers and facilitators for SDM in multidisciplinary sciatica care perceived at different levels of the framework of Grol and Wensing [26]. Both professionals and patients reported more barriers than facilitators. Professionals perceived most barriers at the level of the organizational context, and perceived all facilitators at the level of the individual professional. Patients, on the other hand, reported most barriers and facilitators at the level of the individual professional. It is possible that patients hold the professionals responsible for the care they receive, including the use of SDM, while any barriers on the organizational context that may be important are not visible to them. The professional, on the other hand, is able to see and identify organizational factors as barriers from their perspective, but may also use them as excuses for not having to do anything themselves. This underlines the importance of including both the patient and the professional perspective to identify all barriers for SDM implementation in sciatica. All barriers and facilitators could be classified into a total of 18 themes. A total of 12 themes were the same for patients and professionals and were often related to each other. Patients perceived more facilitators than professionals. This may be due to the fact that professionals have to find a way to integrate SDM during their consultations and have to change their daily practice. Therefore, they may perceive more barriers and fewer facilitators as compared to patients. In addition, most reported facilitators were also reported as barriers, but in the opposite direction.

We found barriers and facilitators corresponding with the literature on uni-disciplinary settings (*e.g*., lack of applicability due to patient characteristics [12], insufficient provider training [28], lack of familiarity about SDM content [12], better patient adherence to treatment [29], motivation [12]). This suggests that barriers and facilitators in uni-disciplinary care also apply to the multidisciplinary setting. Barriers reported in the literature specific to an IP approach and also mentioned in our study are an imbalance of power between health professionals of different disciplines, the existence of professional silos, and disagreement about roles and responsibilities between different disciplines [15].

This study adds barriers and facilitators specifically related to the multidisciplinary context to the literature. These identified barriers and facilitators, include the themes of poor logistics/ implementation, (lack of) sufficient inter-professional collaboration, and reimbursement in favor of surgery. A specific barrier in the theme of poor logistics/ implementation is for instance the conflicting information or advice received from different professionals, so that patients did not know which option to choose. Most patients had visited a GP, physical therapist, and neurologist by the time they visited a surgeon. All of these disciplines have different backgrounds and education, and focus on different aspects of sciatica care, but still it is important that they provide unambiguous information. Regarding lack of inter-professional collaboration, professionals mentioned lack of visibility into what other disciplines can do, and lack of trust in the expertise of other medical disciplines. These barriers cause professionals to talk only about the treatment option they can provide themselves. This may conflict with information given by others. A (better) collaboration and communication between disciplines, and a structure in the information process is necessary (*i.e*., Which professional explains what in which phase of the care trajectory?). To prevent professionals from wasting their time by repeating information from the previously visited professional, it is important that they know what information has already been given to the patient, so that they will have time to integrate SDM in their consultation. As in other studies [12], lack of time was a frequently mentioned barrier for SDM [12]. Structuring the information process ensures that professionals provide sufficient information to the patient within a limited time frame. Furthermore, barriers related to reimbursement in favor of surgery hinder SDM implementation, *e.g*., lack of reimbursement for physical therapy, and financial compensation for sciatica surgery. The reimbursement for surgery is higher than the actual costs, and therefore of financial interest to hospitals. Some surgeons reported that they were encouraged by the hospital to perform surgeries, for example by reserving operating rooms for sciatica surgeries, or even allotting a small amount of money for every sciatica surgery doctors perform. In addition, many private clinics arise because of this reimbursement. These (perverse) incentives may influence the decision making in favor of surgery. On the other hand, physical therapists have a financial interest as well, because they are paid for each treatment. This may cause physical therapists to keep treating the patient instead of referring him or her (back) to the GP. Further research is needed to determine the role of health insurance in SDM, and how the influence of reimbursement on SDM can be reduced. After all, the costs of sustained conservative treatment will be lower than the cost of surgery for insurance companies.

Besides these barriers related to multidisciplinary care, professionals also mentioned that not all patients are able or willing to decide on their care. However, the majority of patients that participated in the focus groups indicated that they do want to decide themselves. The establishment of patient’s preference for his or her role in decision making [30] is an important part of the SDM process, and makes it clear what the patient and professional can expect from each other. Even if they decide jointly that the professional makes the final decision, it still is a shared decision.

A strength of this study is the use of purposive sampling to capture a broad range of perspectives reflecting a diversity of views. We applied purposive sampling by selecting participants from regions with respectively low and high surgery rates, and continued interviewing until data saturation was reached. The participating professionals covered a wide range with respect to age, experience, and number of patients treated annually, so that we can expect that most barriers and facilitators will have been captured by this group. A limitation of this study is the recruitment of patients. Patients were recruited in only one region responding to an advertisement; it is possible that this has caused over-reporting of barriers and facilitators because participating patients were motivated to give their opinion. In addition, patients in other regions, or patients who did not respond to the advertisement, may perceive other barriers or facilitators. On the other hand, participants of the focus groups differed in age, gender and ethnicity. They were also treated in different practices and (types of) hospitals, which ensures variety in perceived barriers and facilitators. A second limitation is the use of quantitative counts within this qualitative study. We reported all barriers and facilitators in tables, but only discuss those barriers and facilitators reported in at least eight interviews or two focus groups, without suggesting that other barriers or facilitators are less important. Based on this study, we cannot determine which barriers and facilitators are the most important barriers or facilitators for implementation of SDM, or how these are associated with characteristics of patients and professionals. Therefore, in the next phase of this study, we will carry out a quantitative study to determine which barriers and facilitators mentioned in this qualitative study are the most important for the adoption of SDM, and professionals’ behavior towards SDM and differences in most important barriers and facilitators between these groups will be determined.

Despite these limitations, our study generated new knowledge that can be used to improve SDM implementation for sciatica patients in the Netherlands and in other countries with a similar context. Furthermore, our study can be used as an example for other patient groups receiving multidisciplinary complex care, given that most perceived barriers by professionals were organization-specific.

## Conclusions

This study provides new insights into barriers and facilitators in a multidisciplinary setting, in primary and hospital care as perceived by both professionals and patients, which is also generalizable for other health problems with multiple disciplines involved. Insight into both barriers and facilitators is essential for the SDM implementation in a multidisciplinary setting. After all, we know from the literature that implementation strategies geared at barriers and facilitators are more effective [31]. Therefore, a multi-faceted strategy is more likely to improve care given to sciatica patients.

## Abbreviations

GP: General practitioner; NL: Neurologist; NS: Neurosurgeon; PT: Physical Therapist; OS: Orthopedic surgeon; P: Patient; B: Barrier; F: Facilitator; SDM: Shared decision making; IP-SDM: Inter-professional shared decision making; ZonMw: The Netherlands organization for health research and development.

## Competing interests

The authors declare that they have no competing interests.

## Authors’ contributions

LB and TV designed the study. SH wrote the article and carried out the study. MW held part of the interviews, coded the interviews and focus groups, and was observator during the focus groups. LB and PM supervised the study, and supervised writing of the manuscript. All authors have critically read and modified both the study protocol and previous drafts of the manuscript, and have approved the final version. All authors read and approved the final manuscript.

## Supplementary Material

Additional file 1 Topic list professionals. Click here for file

Additional file 2 Topic list focus groups. Click here for file

Additional file 3: Table S1 Barriers for SDM according to professionals. Click here for file

Additional file 4: Table S2 Barriers for SDM according to patients. Click here for file
