# Supplementary material for: Barriers and facilitators to implement shared decision making in multidisciplinary sciatica care: a qualitative study
Source: Implement Sci. 2013 Aug 23;8:95. doi: 10.1186/1748-5908-8-95 (PMC3765956; doi:10.1186/1748-5908-8-95)
Supplement: Additional file 1 — Topic list professionals. [file 1748-5908-8-95-S1.doc]

**Topic list professionals**

1. Introduction

Introduction interviewer

Background study

Information about the interview

Introduction participant: profession, years of experience, number patients with sciatica per year, way of working (concerning sciatica), preferences

2. Which factors influence the use of SDM in sciatica treatment?

Topics:

SDM

Prompts:

- Concept SDM – Do they know what SDM is?
- Explanation of SDM: In SDM, clinicians and patients make decisions jointly, weighting the evidence regarding different treatment options [8]. In sciatica care, this means that patients are encouraged to consider both conservative and surgical treatment options, to communicate their preferences and help select the best treatment for their situation.
- Can you give an example of SDM in daily practice?
- Attitude towards SDM in sciatica

Applicability of SDM in patients

Prompts:

- Attitude patient toward SDM
- Explanation scientific knowledge
- Questions patients ask
- Do patients want a more active role?

Requirements SDM

Prompt:

- Decision aid

Environment

Prompts:

- Role (other) professionals/ organization/ colleagues
- Multidisciplinary deliberation
- Factors/ policies at local/ national level

3. What are your recommendations for improving the implementation of SDM in sciatica treatment?
