# Supplementary material for: Barriers and facilitators to implement shared decision making in multidisciplinary sciatica care: a qualitative study
Source: Implement Sci. 2013 Aug 23;8:95. doi: 10.1186/1748-5908-8-95 (PMC3765956; doi:10.1186/1748-5908-8-95)
Supplement: Additional file 2 — Topic list focus groups. [file 1748-5908-8-95-S2.doc]

**Topic list focus groups**

1. Introduction

Introduction moderator and observer

Background study

Information about the focus group

Introduction participant: when diagnosed with sciatica, their care trajectory

2. How was the decision for treatment made?

Topics:

Awareness of treatment options

Information about treatment options

Own preference

Treatment of own preference?

3. Positive and negative experiences

- Explanation SDM: *In SDM, clinicians and patient make decisions jointly, weighting the evidence regarding different treatment options [8]. In sciatica care this means that patients are encouraged to consider both conservative and surgical treatment options, to communicate their preferences, and help select the best treatment for them.*
- Patients are asked to write their positive and negative aspects about the decision making process on post-its, and post their negative aspect on a ‘negative’ board and their positive aspect on a ‘positive’ board.

4. Questions and discussion based on the post-its
