# Supplementary material for: Barriers and facilitators to implement shared decision making in multidisciplinary sciatica care: a qualitative study
Source: Implement Sci. 2013 Aug 23;8:95. doi: 10.1186/1748-5908-8-95 (PMC3765956; doi:10.1186/1748-5908-8-95)
Supplement: Additional file 3: Table S1 — Barriers for SDM according to professionals. [file 1748-5908-8-95-S3.doc]

**Table S1.** Barriers for SDM according to professionals

| **Level** | **Barriers** | **Facilitators** |
| --- | --- | --- |
| Innovation (SDM) | Unclear concept of SDM  **Definition of SDM unclear (when is it truly shared?)** |  |
| Individual professional | Poor professional-patient relationship  **Poor quality of professional-patient relationship**  Negative professional’s attitude/ behavior toward SDM  Lack of interest in sciatica  **Importance to express your own view**  No place for SDM in routines/ habits  No need for SDM  **Preference for one of the treatment options***  Financial interest in one of the treatment options  Lack of knowledge of professional about SDM/ treatment options  **Lack of skills for SDM**  **Lack of education on SDM**  Lack of knowledge on SDM  Lack of knowledge about treatment options  **Lack of knowledge about the sciatica guideline**  Different interpretations of scientific literature leading to different opinions | Positive professional’s attitude/ behavior towards SDM  Motivation (professionals also want a shared decision if they were suffering from sciatica)  Importance of SDM  SDM is essential for good healthcare  SDM improves quality of care  SDM leads to improved patient outcomes/ compliance |
| Patient | Negative patient’s attitude toward SDM/ Lack of patient’s capabilities to decide  Inability of patient to make the decision about treatment  Inability of patient to remember the information given during a visit  Misinterpretation of information by patient  **Patient’s unwillingness to decide**  Pressure by patient toward professional  **Demanding patient**  Pressure for quick recovery of patient  Expectations of patient when visiting a sciatica pathway for having an MRI and surgery |  |
| Social context | Lack of inter-professional collaboration  Lack of communication with other medical disciplines  **Lack of communication between medical professionals and paramedical professionals**  Power struggle between professionals  **Lack of trust in expertise other disciplines**  Lack of interest in other disciplines  Social influences of third parties  Value of repeat consultations underestimated by colleagues  Lack of encouragement from the professional group to apply SDM  Promotion of one of the treatment options third parties* |  |
| Organizational context | Lack of tools to facilitate SDM  Lack of financial compensation for multidisciplinary deliberation  Lack of financial compensation for SDM  Lack of tools to inform patients  Situational factors  **Lack of time during consultation**  Lack of opportunity for a repeat consultation  **Financial interest practice/ hospital/ need for production**  Lack of encouragement from the institution to apply SDM  Long waiting list influences decision process  **Long waiting list for a visit to hospital**  A large/ small number of patients on the professional’s waiting list / under treatment*  Poor logistics/ implementation  SDM is not my task  **Lack of clear criteria for referral and/ or surgery**  **Lack of visibility into what other disciplines can do**  Lack of clear policy for PT's  Lack of agreement about the content and the timing of information provision in the care trajectory |  |
| External environment | Environmental influences on the decision process  Preference for referral to private clinic/ clinic in another country  Availability of treatment options in the area  **Unreliable and contradictory information about treatment options on the internet**  Reimbursement in favor of surgery **Additional payment for physical therapy not covered by insurance**  Waiting list mediation by health insurer  Agreements with health insurance  Competition in Dutch healthcare |  |

*Note.* Barriers indicated in bold were reported in at least eight interviews.

* Two separate barriers
