# Supplementary material for: Barriers and facilitators to implement shared decision making in multidisciplinary sciatica care: a qualitative study
Source: Implement Sci. 2013 Aug 23;8:95. doi: 10.1186/1748-5908-8-95 (PMC3765956; doi:10.1186/1748-5908-8-95)
Supplement: Additional file 4: Table S2 — Barriers for SDM according to patients. [file 1748-5908-8-95-S4.doc]

**Table S2.** Barriers for SDM according to patients

| **Level** | **Barriers** | **Facilitators** |
| --- | --- | --- |
| Innovation (SDM) |  |  |
| Individual professional | Professional-patient relationship  Poor quality of professional-patient relationship  Lack of empathy of the professional  Lack of attention for patient’s personal situation  Lack of attention for patient’s anxiety  **Lack of attention for patient’s preferences**  Lack of confidence in the professional  Negative professional’s attitude/ behavior towards SDM  PT keeps treating the patient without results because of belief in own treatment  **Preference of professional for one of the treatment options***  Lack of guidance in conservative treatment by the professional  Lack of information provision/ explanation  Lack of explanation about the diagnosis sciatica by the professional  **Lack of information provision about treatment options and potential harm and benefits**  **Lack of explanation of the professional about the care trajectory**  Lack of knowledge of the professional about SDM/ treatment options  **Wrong diagnosis by professional**  **Lack of knowledge of the professional** | Professional- patient relationship  A good professional-patient relationship  Attention for patient’s preferences  Attention for patient’s personal situation  GP knows patient’s background  Positive professional’s attitude/ behavior toward SDM  Guidance by the professional  Monitoring recovery  Sufficient information provision/ explanation  Sufficient information provision of the professional  Explanation about harms and benefits of each treatment  Explanation of the MRI images  Explanation of the care trajectory  Openness of professional  Explanation of outcomes scientific research |
| Patient | Negative patient’s attitude toward SDM/ patient’s capabilities to decide  Difficulty to remember everything told during a visit  Anxiety to express own preferences  Lack of confidence in own choice  Anxiety to contradict the professional  Lack of knowledge of patient about treatment options  **Lack of knowledge of patients about one of the treatment options*** | Positive patient’s attitude toward SDM/ patient’s capabilities to decide  **Motivation (important to decide about your own body)** |
| Social context | Lack of inter-professional collaboration  **Lack of communication between professionals**  Lack of trust between professionals  Social influences of third parties  Social pressure of family/ friends on patients | Sufficient inter-professional collaboration  **Communication between professionals** |
| Organizational context | Lack of tools to facilitate SDM  Conflicting information in leaflets  Situational factors  Lack of time during a consultation  Lack of possibilities to discuss problems  Long waiting list influences decision process  **Long waiting list for a visit to the neurologist/ for surgery***  Conflicting information about treatment options  **Conflicting information of the professionals** | Sufficient tools to facilitate SDM  Accessibility of the professional to change treatment  Telephonic consultation  Short waiting list influences decision process  Short waiting list/ quick referral |
| External environment | Environmental influences on the decision process  Pressure of employer for quick recovery  **Unreliable and contradictory information on the internet**  Reimbursement in favor of surgery **Additional payment for physical therapy not covered by insurance**  Individual mandate for a visit to a specialist | Environmental influences on decision process  One reliable website about sciatica |

*Note.* Barriers indicated in bold were reported in at least two focus groups.

* Two separate barriers
